# Presenting a New Framework to Improve Engagement in Physical Activity Programs for Children and Adolescents With Social, Emotional, and Behavioral Disabilities

**DOI:** 10.3389/fpsyt.2022.875181

**Published:** 2022-05-06

**Authors:** April B. Bowling, Jean A. Frazier, Amanda E. Staiano, Sarabeth Broder-Fingert, Carol Curtin

**Affiliations:** ^1^School of Health Sciences, Merrimack College, North Andover, MA, United States; ^2^Department of Psychiatry, University of Massachusetts Chan Medical School, Worcester, MA, United States; ^3^Eunice Kennedy Shriver Center, University of Massachusetts Chan Medical School, Worcester, MA, United States; ^4^Department of Pediatrics, University of Massachusetts Chan Medical School, Worcester, MA, United States; ^5^Pennington Biomedical Research Center, Louisiana State University, Baton Rouge, LA, United States; ^6^Department of Family and Community Medicine, University of Massachusetts Chan Medical School, Worcester, MA, United States

**Keywords:** exercise, mental health, pediatrics, psychiatric, neuro developmental, implementation science

## Abstract

Children and adolescents with psychiatric and neurodevelopmental diagnoses such as anxiety, depression, autism, and attention-deficit/hyperactivity disorder (ADHD) face enormous health disparities, and the prevalence of these disorders is increasing. Social, emotional, and behavioral disabilities (SEBD) often co-occur with each other and are associated with unique barriers to engaging in free-living physical activity (PA), community-based exercise and sports programming, and school-based physical education. Some examples of these barriers include the significantly depleted parental reserve capacity associated with SEBD in children, child dysregulation, and previous negative experiences with PA programming and/or exclusion. Importantly, most SEBD are “invisible,” so these parents and children may face more stigma, have less support, and fewer inclusive programming opportunities than are typically available for children with physical or intellectual disabilities. Children’s challenging behavioral characteristics are not visibly attributable to a medical or physical condition, and thus are not often viewed empathetically, and cannot easily be managed in the context of programming. Existing research into PA engagement barriers and facilitators shows significant gaps in existing health behavior change (HBC) theories and implementation frameworks that result in a failure to address unique needs of youth with SEBD and their parents. Addressing these gaps necessitates the creation of a simple but comprehensive framework that can better guide the development and implementation of engaging, effective, and scalable PA programming for these youth and their families. Therefore, the aim of this article is to: (1) summarize existing research into SEBD-related child and parent-level barriers and facilitators of PA evidence-based program engagement; (2) review the application of the most commonly used HBC and disability health theories used in the development of evidence-based PA programs, and implementation science frameworks used in adaptation and dissemination efforts; (3) review the SEBD-related gaps that may negatively affect engagement; and (4) describe the new Pediatric Physical Activity Engagement for Invisible Social, Emotional, and Behavioral Disabilities (PAID) Framework, a comprehensive adapted PA intervention development and implementation adaptation framework that we created specifically for youth with SEBD and their parents.

## Introduction

Social, emotional, and behavioral disabilities (SEBD) encompass both neurodevelopmental and psychiatric diagnoses and their attendant symptoms that significantly impair activities of daily living and learning. These diagnoses include, but are not limited to, autism, attention-deficit/hyperactivity disorder (ADHD), depression, and anxiety disorders, and they have high rates of co-occurrence (1–3). Between 2000 and 2011, the prevalence of children needing school and medical accommodations for these neurodevelopmental and mental health conditions increased by 21% in the United States (4). Importantly, these conditions often present as “invisible,” meaning that their behavioral symptoms are not visibly attributable to a medical or physical condition or disability. This means that these children and their families often do not receive the support and inclusion they need (5, 6) and are often mischaracterized by others as “bad,” “lazy,” “uncooperative,” or “undisciplined.” Likewise, their parents and caregivers are often implicated as responsible for their child’s problematic behaviors (7, 8). The stigma associated with such judgments creates an extra barrier to engagement and inclusion for these youth and their families across a variety of programs and settings.

Physical activity (PA) is critical for healthy cognitive and physical development in children and adolescents, and there is mounting evidence that exercise is “medicine” for youth with social, emotional, and behavioral disabilities (SEBD) (9, 10). Unfortunately, studies have found that PA levels among youth with SEBD tend to be significantly lower than peers without these disabilities. Research indicates that autism and depression are associated with participation in fewer types of PA and with more sedentary behaviors (11, 12). In addition, youth with ADHD and autism get significantly less PA each day than their typically developing counterparts (13, 14). Sport participation is also lower for youth with a variety of SEBD (15). These low levels of PA participation are highly problematic, contributing to the health disparities experienced by those with SEBD including high risk of obesity and multi-morbidity.

The physical health benefits of PA, which include planned exercise as well as general movement and free play, are well documented (16). Evidence of PA’s causal effects on mental health including brain development, cognition, and emotional regulation are also mounting (17–20). For example, a systematic review of PA interventions in children with heterogeneous SEBD found that both acute (single-bout) and chronic (repeated bouts over time) PA resulted in improvements in a variety of disability-related outcomes (21). For example, chronic aerobic exercise was associated with improvements in sociability and classroom functioning in children with autism and ADHD (21, 22). Acute and chronic aerobic exercise were also found to have positive effects on executive function and objective neurological outcomes, and frequent aerobic exercise has been found to have positive effects on mood in children with mood disorders and/or ADHD (21). While research continues to elucidate the relationships between different modalities of PA (e.g., free play, aerobic exercise, high intensity interval training, strength training, yoga) and their role in a variety of cognitive, mental, and physical health outcomes, interventions and evidence-based programs to improve PA in children and adolescents have proliferated (23, 24). Such interventions and evidence-based programs to improve PA in youth are hereafter jointly referred to as PA programming.

Despite advances in evidence-based PA programming for children in general, development and effective implementation of PA programming for the pediatric population who may need them most – those with SEBD – have lagged. Rimmer and Vanderbom (25) suggested that a worthy approach to the problem would be an increased emphasis on inclusion team science – i.e., bringing together PA intervention designers with disability health experts and individuals with lived experience to adapt existing PA evidence-based programs more rapidly for children with disabilities. However, even theoretically guided adaptations of existing evidence-based PA programs often fail to engage youth with SEBD and their families in real-world settings (26), where engagement is defined as encompassing both program reach (recruitment response, enrollment, and retention) and participants’ willingness/ability to execute prescribed PA of a specific modality, frequency, duration, and/or intensity (adherence/fidelity and dose received) (27). PA programming engagement is particularly low among youth with SEBD from historically marginalized groups such as those living in poverty and those who are Black, Indigenous and People of Color (BIPOC), or lesbian, gay, bisexual, transgender, or gender queer (LGBTQ+) (28, 29). This is not surprising, given that SEBDs are often invisible, comorbid, and highly stigmatized. This leads to unique and often daunting barriers to PA programming engagement at the child, parent, and community/system levels (26). These barriers include peer exclusion, a lack of inclusive opportunities for youth with behavioral challenges, drained parental emotional reserves and lack of social support, and symptomatic dysregulation in youth that diminishes PA self-efficacy and motivation, to name a few (26). Such barriers may only seem surmountable for families with considerable resources.

Among studies of barriers and facilitators of PA programming in youth with SEBD that reported sample demographics, about 15% were non-white and about 81.5% were male participants, the latter of which aligns with the ratio of males to females diagnosed with autism but does not represent all SEBDs (26). In a review of PA intervention outcomes in youth with SEBDs, few studies reported participants’ race/ethnicity or socioeconomic status, and those that did tended to have samples that skewed toward white and above average income, with high parental education (21). Even among higher resourced families, retention and adherence remained major feasibility challenges in many PA interventions (30), raising questions about dissemination and potential reach.

Health behavior change (HBC) theories are used to guide the development of PA interventions and determine their efficacy in controlled settings such as randomized controlled trials in order to determine the final package of a PA evidence-based program (31). Implementation science is then defined as a specified set of activities designed to put into practice a prescribed activity or well-defined program (32), such as an existing PA evidence-based program (33). Implementation science frameworks guide the strategies for adaptation and assessment of evidence-based program replication and dissemination, analogous to how theories of HBC are used to guide the development and testing of the initial intervention. However, given that clear engagement gaps exist for both initial PA intervention development for youth with SEBD, as well as for evidence-based program dissemination to community settings, both translational and implementation scientists need an integrated engagement framework to better meet the needs of diverse children and adolescents with SEBD and their families.

Given that context, the purpose of this paper is to:

1) Summarize existing research into SEBD-related child and parent-level barriers and facilitators of PA evidence-based program engagement;
2) Review the application of the most commonly used HBC and disability theories used in the development of PA evidence-based programs, implementation science frameworks used in their adaptation and dissemination, and SEBD-related gaps that may negatively affect engagement; and
3) Describe a new PA engagement framework specifically for youth with SEBDs, which can be used to help design interventions or be embedded into existing implementation science frameworks to improve adaptation and dissemination of existing evidence-based PA programs.

While not a systematic review of the literature, this policy and practice review synthesizes existing reviews and major papers from three primary areas to inform development of the PA engagement framework for youth with SEBDs. Those areas are (1) existing research on barriers and facilitators to PA engagement specific to these youth and their families, (2) health behavior and disability health theories used to design PA interventions, and (3) implementation frameworks used to implement and evaluate evidence-based PA programming. Area 1 is discussed based on our recent systematic review of the literature which was published in 2021 (26). Areas 2 and 3 are based on existing review and synthesis articles by experts in relevant health behavioral theory (27, 34) and implementation science domains (27, 35, 36). A detailed discussion of the literature is contained in the relevant sections that follow.

## Social, Emotional, and Behavioral Disabilities-Related Barriers to Physical Activity Evidence-Based Program Engagement in Children and Adolescents

Unlike adult engagement in in PA interventions, children’s and adolescents’ engagement is significantly mediated by parent and caregiver (hereafter simplified to “parent”) support and actions. While community barriers to PA engagement among youth with SEBD and their families are significant and include lack of availability, no SEBD-specific staff training, safety issues, and inappropriate environmental features (e.g., loud gymnasiums) (26), barriers most specific to recruitment, retention, and adherence center on parent/caregiver and child-level characteristics which are the focus of this article. Our recent review of 24 studies investigating barriers and facilitators of physical education, sport, and PA program participation among children and adolescents with heterogeneous and often co-occurring neurodevelopmental and psychiatric disabilities cataloged these factors (26), which are reviewed by category in context with existing literature below.

### Child Level Factors

The most commonly cited child-level barriers to PA programming engagement include low-motivation, social isolation, peer exclusion, dislike of competition, preference for solitary activity, motor delays, sensory and/or behavioral dysregulation, and medication side effects (26, 30). Even if SEBD-specific PA programming is adapted to address these concerns, children’s previous PA-related experiences in school PE classes, community PA programming (e.g., swim classes), and recreational sports are likely to affect – in many cases negatively – their outcome expectations associated with future PA engagement (37). Thus, their willingness to enroll and participate in opportunities such as SEBD-specific PA programs is often low. Even more importantly, many evidence-based PA programs targeting youth with SEBD have *not* been specifically adapted to account for these child-level factors. For example, adapted PA programming often takes place in groups due to resource constraints, and therefore may also implicitly include or elicit elements of comparison and competition that can discourage participation and increase behavioral dysregulation (26). Several studies have found that youth with autism without an intellectual disability participate in physical education programming at lower rates, specifically because of fears about locker room behaviors and sensory overload associated with gymnasium-based activities (38–40). Without considering these SEBD-specific barriers, PA programming cannot be truly inclusive and participation will remain low.

### Parent/Caregiver Level Factors

Parenting any child is a challenging experience that draws on a variety of individual and familial resources. Parental competencies, innate personality characteristics such as optimism and patience, internal resources such as self-efficacy and ability to manage stress, and external resources such as social support and financial resources are important factors influencing parenting behaviors, including those relevant to encouraging their child’s PA-related behaviors and engagement in PA programming (8). A recent review found that the most important parent-level determinants of PA programming engagement when a child has a SEBD include depleted caregiver reserves, parent education level, financial resources, and caregiver support for and participation in and positive attitude toward PA (26). In particular, depleted parental reserve capacity – defined as the drawing down of combined emotional, social, logistical, and financial reserves – was the most common barrier cited by parents related to their child’s PA programming enrollment and retention (30, 40–44). This is also important because parent participation in a role modeling of exercise engagement is a predictor of children’s PA participation (45), yet parents with excessive reserve capacity depletion are less likely to be willing or able to exercise themselves unless given additional support to do so (e.g., time, encouragement, logistical resources).

Parental reserves also interact with child characteristics to determine parenting choices and resultant behaviors. For example, in order to limit screen time and promote PA, a parent must be willing to set limits, have the ability to enforce those limits, be invested enough to follow through with enforcement if challenged, and have the resources to redirect the child to an alternative activity. If the child is particularly challenging, for example displaying intense oppositional or defiant behaviors, each of those steps requires greater input of parental resources. As might be expected, therefore, studies of parenting children with disabilities have identified several parental resources that give rise to significant additional resource reserve drains, even before considering intersectionality with race/ethnicity, poverty, or other historically marginalized characteristics. In particular, it is well established that parents of children with invisible disabilities experience excessive (1) chronic stress that drains internal resources; (2) logistical, time, and financial drain from obtaining required services and addressing co-morbid conditions in their children; and (3) depletion of social capital through a variety of child and family support pathways (46, 47). A meta-analysis of studies comparing the experience of stress in parents of children with and without autism found that parenting a child with autism resulted in higher scores on multiple stress measures for both mothers and fathers. Stress was greatly increased as compared to parents of typically developing children (mean effect size 1.58, *p* < 0.001), but also higher than in parents of children with visible disabilities like Down syndrome (mean effect size 0.64, *p* < 0.001) (8). In their study of stress in parents with disabled children, Dumas et al. found that children with clinically identified behavioral disorders had parents who experienced statistically and clinically higher levels of parenting stress than parents of typically developing children as well as those with other types of disabilities, and that mothers of this group in particular presented with significantly higher levels of depression (46). These higher rates of depression appeared to be specifically related to stressors of parenting a behaviorally challenging child as opposed to parental psychopathology (46, 47). Social isolation, less institutionalized support for these types of disabilities, emotional drain due to parenting challenging or worrisome behaviors, and marital discord related to parenting stressors are just a few of the identified pathways through which stress and associated mental health impacts are amplified in parents of behaviorally disabled children (8, 47, 48).

**FIGURE 1:**
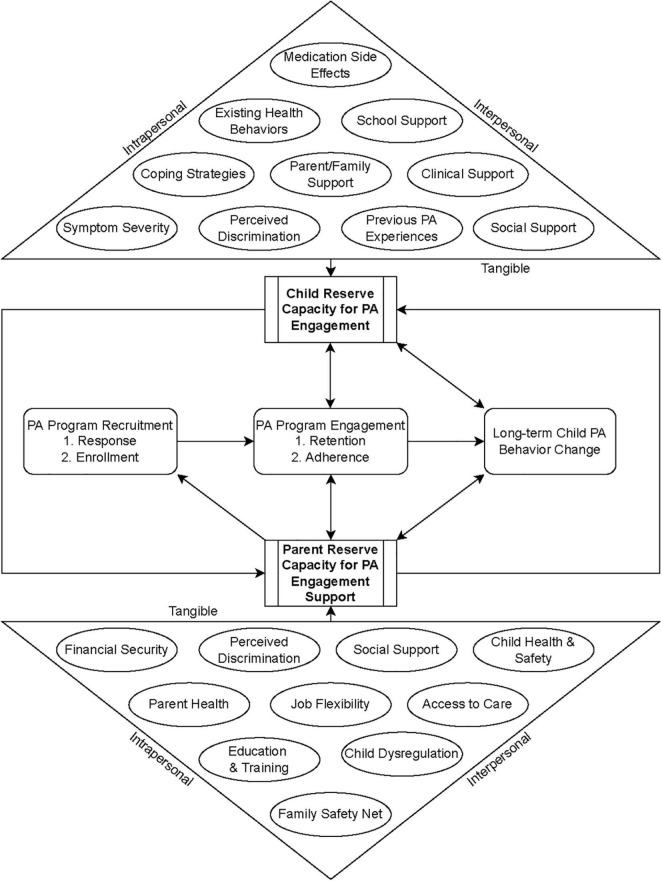
Conceptual model of pediatric SEBD and PA programming engagement.

Figure 1 shows a conceptual model of how child and parent reserve capacity interact and mediate the child’s recruitment, retention, and adherence to PA programming when a child has a SEBD and potentially other types of invisible disabilities. Definitions of the intrapersonal, interpersonal, and tangible factors shown in the figure as affecting reserve capacity are contained in Table 1 and are not exhaustive. PA, like many health behaviors, has a bidirectional association with aspects of reserve capacity, such as emotional and social capital (49). For example, PA requires motivation and logistical support to initiate and complete but improves emotional regulation and sociability after engagement (49). Likewise, as discussed previously, parental and child reserves and associated emotional and behavioral states are reciprocal and impact each stage of PA programming engagement within context of one another. While this paper’s specific focus is on PA engagement in children with SEBD, it is important to note that parenting encompasses competing priorities, of which a child’s PA is only one health-related concern that parents are compelled to address. Parents must additionally direct resources toward managing the other chronic health problems associated with these disabilities, as well as addressing educational, social, emotional, and behavioral goals and long-term care arrangements (6, 46, 47). This heightened resource demand becomes increasingly significant for families with intersectional, marginalized characteristics who may fear increased stigma and discrimination and additional barriers to access to care.

**TABLE 1:** Definitions of factors affecting parent and child reserve capacity.

Factor	Definition
Child	
Symptom severity	The severity of SEBD-related disregulation experienced by the child at a given point in time. Can fluctuate and affect child PA perceptions and engagement differently across time.
Medication side effects	Physical effects of psychotropic medications affecting PA engagement, including lethargy and weight gain.
Coping strategies	Non-pharmacologic tools a child feels able to implement to manage their symptoms and impacts on wellbeing.
Perceived discrimination	Perceived stigma, judgment, and exclusion related not only to the child’s SEBD, but also intersectional characteristics such as race/ethnicity, gender identity, and/or sexual orientation.
Previous PA experiences	Positive, negative, or neutral experiences the child has had engaging in PA in previous programs and settings.
Social support	Friend/peer/adult relationships external to the child’s extended family that provide child social support.
Clinical support	Ongoing clinical care that supports child wellbeing and symptom regulation/alleviation.
Parent/family support	Family-based emotional, financial, and logical support for the child. Can include parent/caregivers, siblings, and extended family.
School support	Educational, learning, and social supports provided by academic institutions. These may be human (e.g., teachers and aids) or structural (e.g., accessible learning spaces, individualized educational plans).
Existing health behaviors	Child health habits that affect SEBD symptoms and overall wellbeing, such as sleep, nutrition, screen time, and PA.
Parent	
Financial security	The extent to which the parent has both adequate income to meet family needs, including care for their child with SEBD, and savings to meet unexpected or future financial demands, such as long-term care for their child after the parent’s death.
Parent health	Parent physical and mental health.
Child health and safety	Child physical health and safety, including concerns about suicidality, substance use, and criminal justice risk.
Child disregulation	The severity of SEBD-related child disregulation experienced by the parent at a given point in time. Can fluctuate and affect parent perceptions and willingness/ability to support child PA engagement differently across time.
Perceived discrimination	Perceived stigma, judgment, and exclusion related not only to their child’s SEBD, but also the parents’ own intersectional characteristics such as mental health challenges, race/ethnicity, gender identity, and/or sexual orientation.
Social support	Perceived parental social support.
Job flexibility	The extent to which a parent can set their own hours, take time off, or work remotely to provide care, transportation, or attend appointments with their child.
Access to care	Includes characteristics of health insurance and availability of quality health care for the parent and child.
Family safety net	Parental perceptions of tangible resources available to provide for family needs in the event of financial, health, or other crises (e.g., house foreclosure, job loss, and divorce) in addition to what the parent alone can provide.
Education and training	Level of parent educational attainment and SEBD-related parenting training.

## Theories and Frameworks for Physical Activity Evidence-Based Program Development and Implementation

There are scores of theories of HBC and implementation frameworks, all of which explicitly or implicitly address aspects of participant engagement (27, 35). Although terms vary between theories and frameworks, the constructs of recruitment, enrollment, and retention together are most often referred to as “reach” in implementation literature pertaining to PA evidence-based programs (27). Engagement is alternatively conceptualized as adherence, an aspect of fidelity, and exposure dose (27). Here, we focus on those theories and frameworks applicable and most commonly used to guide develop, implement, and evaluate evidence-based programs for (a) PA interventions, (b) pediatric populations, and (c) individuals with disabilities.

### Relevant Theories of Health Behavior Change

Children and teens with SEBD depend heavily on family support and parent actions to engage in PA. The Family Ecological Model developed by Davison (50) is an ecological framework developed to ensure consideration of child, parent, and community level factors that uniquely impact pediatric engagement in HBC interventions. While not a theory of HBC, the Family Ecological Model posits that parental characteristics heavily mediate children’s engagement in health behavior interventions, even when those interventions are not family-centered. These characteristics must be addressed through tailoring of intervention components to meet the needs of parents in the target population. Davison further used the Family Ecological Model to develop the Family-Centered Action Model of Intervention Layout and Implementation (FAMILI) (51), which is covered under the implementation frameworks section below.

Meanwhile, the four most commonly used theories of HBC – Health Belief, Theory of Planned Behavior, Social Cognitive, and Transtheoretical – were not developed specifically for children or individuals with disabilities. However, Ravesloot et al. (34) identified the cross-theoretical constructs from all four theories and their application for individuals with disabilities within the context of the World Health Organizations International Classification of Function (ICF) framework. Within this framework, PA programming is classified as a self-care activity that influences the course of physical health, ability for activities, and domains of participation. Five common constructs across the four major theories of HBC were identified as reflecting the dynamic interaction of environmental and personal factors described in the ICF for individuals with disabilities: (1) outcome expectations; (2) self-efficacy; (3) social norms; (4) reinforcement management and stimulus control; and (5) environmental facilitators (34). Table 2 lists each construct with its definition, relationship to SEBD-related PA engagement barriers, and examples in practice.

**TABLE 2:** Cross-theoretical HBC constructs and application in SEBD-specific PA programming engagement.

HBC construct (34)	Definition in context with disability (34)	Relationship to child-level SEBD-related PA barriers and facilitators (26)	Examples in practice
Outcome expectations	Beliefs about behavioral choice consequences, including perceived risks and benefits. Risks are often inflated for individuals with disabilities, while benefits may be decreased.	Low motivation	PA is too hard and will make me feel worse. PA is boring.
		Social isolation/peer exclusion	I will get left out by others during this programming.
		Dislike of team/group activities, and preference for solitary activities	This programming will be competitive even if they say it isn’t, just like PE class.
		Structure, predictability, and consistency	I know what I will be expected to do during this programming.
		Opportunities for paired/group and solo PA	I know I can do the PA alone or with a friend depending on how I feel.
Self-efficacy	Beliefs about one’s ability to change behavior and control events in one’s life. Often reduced in individuals with disabilities. This reduction is exacerbated in children and teens, who have reduced agency.	Low motivation	I’ll never be athletic so why would I go?
		Motor skills difficulties	I’m always the worst at sports and I never get better.
		Preference for solitary activities	No one can see how bad I am at this PA while I work on getting better.
		Sensory/behavioral dysregulation	I will have a quiet space to practice PA so I can pay attention to my instructor.
		Medication side effects	My medication makes me too sleepy to go my PA program.
Reinforcement management and stimulus control	The occasion for performing a behavior, cues to action, and rewards for taking action. Individuals with disabilities often have reduced occasions to perform a behavior (fewer opportunities). Cues to action and rewards must be specific to individuals with disabilities.	Low motivation	If I go, I get to pick the type of PA I want to practice this week. My coach texted me a reminder that I will feel better after going for a walk today, even if it is short and easy.
		Structure, predictability, and consistency	If I practice, I will receive the reward I have chosen.
Social norms	Beliefs about social approval/disapproval of performing a given behavior. Individuals with disabilities are often presented with different norms, which leads to internalizing societal expectations for worse health behaviors and poor health outcomes.	Low motivation	My friends and I are video gamers, not jocks; we don’t do PA.
		Behavioral dysregulation	People like me don’t go to programs like that.
		Medication side effects	I’ve gained too much weight; heavy people don’t exercise.
Environmental facilitators/barriers	Features of the physical or programming environment that encourage or discourage a behavior. Often underappreciated in the case of individuals with invisible disabilities.	Sensory/behavioral dysregulation	The gym where we practice is too loud, and I get overwhelmed and anxious. I’m not taking another long bus ride after school when I’m already exhausted and feeling anxious about all the homework I have to do.

In the context of youth with SEBD, outcome expectations for PA evidence-based program may be particularly low, given previous PA engagement experiences and self-efficacy that may be affected by gross and fine motor delays, sensory dysregulation, and attentional challenges (30). Social norms may skew toward sedentary activities (i.e., video games versus high school sports), while reinforcement management and stimulus control may have increased importance due to symptom constellations and perceived lack of agency in previous PA programming such as PE classes. Finally, environmental facilitators may be under-emphasized for youth with SEBD compared to those with physical or intellectual disabilities. While accommodations may be less obvious, studies have found PA engagement in these youth is mediated by a variety of environmental factors such as noise and brightness, transport time, and perceived safety (26).

In the field of disability health, Empowerment Theory posits HBC is most possible when individuals with disabilities are empowered to be active participants in and have personal control over their own lives, and when those around them – caregivers, service providers, and programs – possess the requisite knowledge, skills, and tools to provide effective services and support (52). The Empowerment Model (53) is an intervention design and evaluation framework that has been used for PA promotion among youth with physical and intellectual disabilities (54). This model encompasses three key constructs: support, training, and programming’s continuum of opportunity (54). Support is conceptualized as the adaptation and provision of resources to participants that are specific to their disability-related needs, provision of resources to enable parent support of the child with a SEBD, and the provision of resources to support inclusive programming and policy implementation at the community level. Support includes education but cannot be limited to it, and must include increased resources and infrastructure. Training refers to the process of creating child, parent and interventionist/staff capacity to use equipment, systems, and processes that empower engagement and promote inclusion. Finally, the continuum of opportunity refers to the intersection of setting and group composition (i.e., specialized to the disability group only; inclusive of the disability group; reverse-inclusive with those without the disability), which is an important intervention design consideration that helps determine the support and training necessary to implement the adapted intervention.

Although the Empowerment Model explicitly addresses the need for support in PA programming, none of the most commonly used HBC theories explicitly address impact of families experiencing increased drains on emotional, social, logistical, and financial reserves associated with invisible disabilities such as SEBD, which have been found to be the most important determinants of PA programming engagement for families of children with SEBD. However, the model of Reserve Capacity developed by Gallo and Matthews (55) acknowledges that individuals facing significant burdens such as poverty or parenting a child with a disability (or both) experience excessive resource drains that leave them vulnerable to health disparities (56). Reserve capacity has been found to be the aggregate of optimism, self-esteem, and social support that is inversely related to development of negative emotions including depression, anger, and tension, and mediates the relationship between socio-economic status (SES), care engagement, and a variety of cardiometabolic outcomes (56, 57). Where SES represents less access to resources and lower position in the social hierarchy in the original tests of the Reserve Capacity Model, these two characteristics are also clearly associated with disability (58, 59).

As a theoretical construct, reserve capacity encompasses the personal resources of an individual, such as perceived control, self-esteem, and optimism, as well as the social resources of that individual, such as social integration, social capital, and financial resources. It can additionally be seen to include psychological resources, such as emotional energy. As previously discussed, these factors affect parenting of PA-related health behaviors in children with behavioral challenges. Adequate reserve capacity is, therefore, required to parent any child effectively, including parenting around children’s health behaviors. Parents experiencing excessive drains of reserve that are unaddressed will be less capable of mustering the resources necessary to parent health behaviors such as PA successfully.

Because of the competing health priorities their children face, and the associated reserve capacity drains they experience, parents of children with SEBD may be particularly inclined to view their children’s lifestyle behaviors as a secondary concern, especially if clinicians are not directly addressing the importance of exercise during appointments. Parents may interpret clinicians’ lack of engagement on nutrition and exercise behaviors, or emphasis on BMI assessment alone, as reasons to direct less of their already thinly spread reserves toward addressing those behaviors seen as only affecting weight such as screen time, PA levels, and diet. Unfortunately, this may have the unintended consequence of worsening the behavioral dysregulation of the child and further diminishing parental reserves, due to the often unrecognized but potentially important effects that lifestyle behaviors such as PA have on cognitive and behavioral functions.

### Relevant Implementation Frameworks

A recent study of implementation frameworks used in PA and behavioral nutrition interventions found that the Framework for Effective Implementation (36) and Consolidated Framework for Implementation Research (35) were most commonly used, and recommended a minimum data set of implementation determinants and outcomes for use by PA evidence-based program researchers based on an expert panel’s selection process (27). On the 5-stage scale-up continuum (27), engagement among diverse participants is generally not a focus of formative evaluation (stage 1) or efficacy studies (stage 2), which often depend on biased sampling (recruitment) methods that result in mainly white participants from two-parent middle and upper-middle class households. Instead, problems with adherence and reach among diverse participants generally emerge during real world trials (stage 3) and dissemination efforts (stage 4), when the focus of implementation evaluation is the extent to which the intervention is delivered to the target population as planned (27). This reality is problematic, given that reach is the most highly ranked indicator of implementation success by experts, followed closely by adherence (defined as dose received and fidelity) (27). This indicates a need to focus more on engagement in initial intervention design and efficacy testing, and a more specific framework to guide adaptation implementation strategies affecting engagement during real world trials and dissemination. While the Framework for Effective Implementation includes participant responsiveness and reach as key aspects of implementation and relates them to characteristics of the innovation (adaptability and compatibility) and factors relevant to the delivery system (shared decision-making regarding adaptations) (36), there is no clear guidance on how responsiveness and reach would be achieved in the complex case of PA evidence-based programming for youth with SEBD. The Consolidated Framework for Implementation Research differentiates between the core components of an evidence-based program and the “adaptable periphery” that should be tailored to meet the needs of specific target populations depending on the implementation setting (35). In the case of PA evidence-based program, the PA duration and intensity dose may be considered core, while the modality and delivery system may be considered adaptable periphery. However, it is unclear how much of an initial evidence-based program can be altered to improve reach and adherence, such as adapting setting or delivery to accommodate the needs of families of children with SEBD, and how much must be retained in the program for it to still be considered evidence based. Therefore, including youth with SEBD and their caregivers earlier in the process including in the initial formative and efficacy studies as well as during adaptation of existing PA programs is critical for successful dissemination and implementation.

Finally, Davison et al. put forth the FAMILI to help guide development and implementation of family-centered obesity prevention programs (51). FAMILI recognizes that parents play a fundamental role in shaping children’s lifestyle behaviors, including diet and PA, but that most interventions targeting children do not either directly include parents or focus on sustainable change at the level of the family. Centered on the Family Ecological Model as previously described, FAMILI presents three phases for family-centered intervention and programming implementation, regardless of intervention setting or target: (1) use theories of family development to frame family-centered research and practice; (2) use a mixed-methods approach to examine factors affecting families that are relevant for intervention design; and (3) use participatory methods to develop, implement, and evaluate family-centered interventions that empower parents to establish healthy family lifestyles (51). While broad, FAMILI is an important frame for new interventions and programs aiming to improve PA in youth with SEBD, whether they occur at home, in school, in clinical settings, or in community settings. And while it may not be fully feasible when adapting existing evidence-based PA programming for youth with SEBD, all three phases should be considered during the adaptation process. However, a more SEBD- and PA-specific framework that incorporates the general FAMILI phases is necessary to practically guide programming development, adaptation, implementation, and evaluation for these youth and their parents as FAMILI does not specifically address parental reserve capacity.

## Actionable Recommendations: The Paid Framework

**FIGURE 2:**
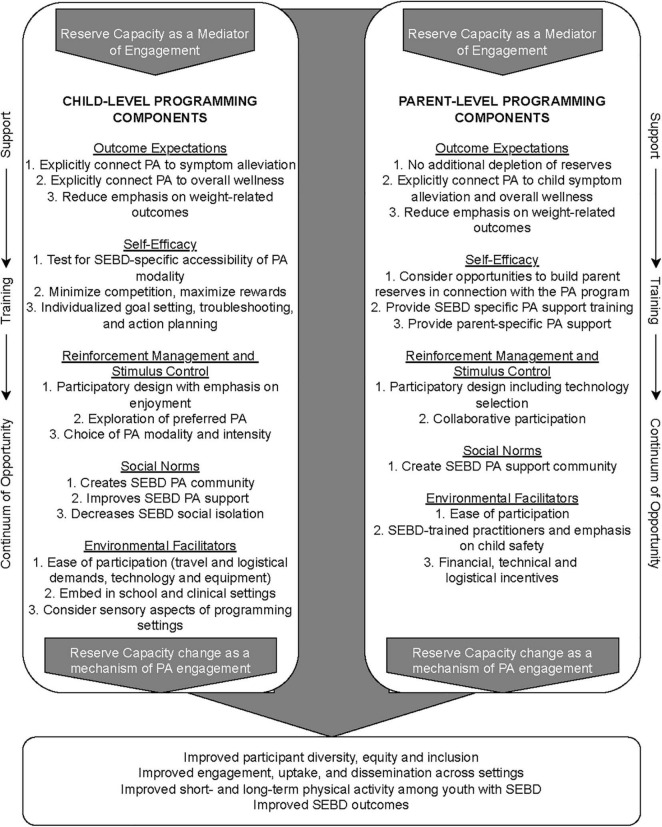
PAID Framework.

It is clear that while many established theories and frameworks may have applicable constructs, a simple and specific framework is needed to improve PA programming engagement among youth with SEBD. Such a framework must: (a) help overcome pre-existing PA outcome expectations of youth with SEBD and their caregivers; (b) incorporate the mediating role of parents in pediatric PA programming; (c) address uniquely depleted reserve capacity in both youth and parents due to SEBD; (d) be theory-based; and (e) fit within existing, commonly used implementation frameworks such as the Consolidated Framework for Implementation Research, Framework for Effective Implementation, and FAMILI. We developed the Pediatric Physical Activity Engagement for Invisible Social, Emotional, and Behavioral Disabilities (PAID) Framework (Figure 2) to address the SEBD-specific barriers and facilitators of PA programming engagement identified in the literature using disability-specific HBC theoretical constructs. PAID may be used together with an overarching HBC, disability health, or ecological theory during the intervention design stage to improve the diversity of participants during formative and efficacy research. Conversely, PAID can also be nested within existing implementation frameworks such as Consolidated Framework for Implementation Research, Framework for Effective Implementation and FAMILI to improve engagement in an existing PA evidence-based program.

Because PAID is geared toward improving PA programming participation, and such engagement in children is both predicated on (e.g., for home-based or community-based programs) and mediated by (e.g., for school-based programs) parent support, the PAID Framework is intentionally grounded in the Family Ecological Model, addressing both child and parent-level factors influencing PA engagement. Likewise, because depleted parent reserve capacity is the most common barrier to pediatric PA intervention reach (recruiting, enrollment, and retention) for youth with SEBD cited in the current literature, PAID places parent-oriented components within the context of explicitly *increasing* reserve capacity, or at the very least, not depleting it further via intensive participation demands. PAID recognizes caregiver and child reserve capacity as both mediators of PA programming engagement as well as important outcome measures as predictors of long-term PA engagement.

Within the context of the Family Ecological Model and augmented by the Reserve Capacity Model, the PAID Framework then maps intervention component design and implementation adaptation considerations to five SEBD and PA-specific HBC theoretical constructs related to intervention engagement as defined in Table 1. These include: (1) outcome expectations; (2) self-efficacy; (3) reinforcement management and stimulus control; (4) social norms; and (5) environmental facilitators. The design and implementation considerations recommended under each construct address the major child and parent-level barriers and facilitators of PA programming engagement identified in the literature.

For example, as previously discussed, youth with SEBD may have particularly negative PA programming outcome expectations, which impact motivation and must be overcome to improve recruiting, enrollment, and retention. Therefore, the PAID Framework explicitly recommends design and adaptation of PA interventions and evidence-based programs that disconnect (i.e., underemphasize) PA from fitness, competition, and weight-related outcomes, and instead connect PA engagement to SEBD symptom improvement and an overall sense of wellbeing, recreation, and enjoyment. These constructs are also clearly inter-related; for example, if interventions feature a participatory design element with an emphasis on PA enjoyment as recommended as an aspect of reinforcement management and stimulus control, that inclusion can also help improve outcome expectations and enrollment of later PA evidence-based program implementation. The outer layer of the PAID Framework indicates that implementation of PA programming should be considered across levels of the empowerment model to promote real world engagement. Therefore, regardless of implementation framework, PA programming implementation strategies for youth with SEBD and their families must address support, training, and continuum of opportunity.

While not explicitly included, consideration of the learning needs of youth with SEBD is needed, since many of these youth may have cognitive or learning disabilities that render traditional instructional approaches within PA programs inaccessible to them. Many PA programs include educational or instructional components that are designed to impart critical information and skills in order to effect skill mastery, behavior change, and ultimately, improved outcomes. Accordingly, universal design for learning (UDL) instructional approaches for youth SEBD present a viable approach for making adaptations for this population. UDL is an evidence-based framework (60–62) whose goal is to maximize program participants’ understanding, expression, and application of learning by providing multiple ways by which they can acquire information and demonstrate their learning and skill acquisition. UDL features three main components: (1) multiple ways to engage youth and making explicit the “why” of learning (for example, providing program participants with choices, building skills through games, and ensuring that activities are perceived to be relevant); (2) promoting comprehension and understanding *via* multiple means of representation (i.e., the “what” of learning; for example, providing information in written, audio, and/or pictorial formats, along with demonstrations and hands-on learning); and (3) addressing the “how” of learning and its application and demonstration by learners in multiple ways (for example, encouraging youth to tell or show what they have learned and providing opportunities for them to demonstrate new skills in a supportive context).

All components may not apply for every PA intervention or evidence-based program, and how they are conceptualized/put into practice will vary based on setting, intended outcome, population, and stage of scale-up. However, certain elements of the PAID Framework are considered essential, particularly a participatory design and adaptation process, as well as inclusion of child and parent reserve capacity as a PA programming assessment measure. This encourages program design that includes components targeting building reserve capacity, instead of drawing upon it. For example, PA programming aimed at children may consider added support for independent parent PA engagement as well, both as a form of self-care and as an opportunity to model positive behavior change for their children. Special care should be taken to ensure that intersectionality is considered in participatory processes and that such activities minimize additional resource drains on youth and caregivers. Finally, while the focus of PAID is on parent and child-level factors affecting PA engagement, it is critical to consider both setting and inclusion spectrum when considering PA design. For example, as previously discussed, setting can influence both parent and child reserve capacity through a variety of mechanisms (transportation requirements, accessibility, etc.); schools are an important context for children’s PA, and programming delivered there does not require additional resource inputs from parents. Likewise, PAID includes Empowerment Model constructs of support, training and continuum of opportunity (54), which are explicitly aimed at community and peer-level influences on PA engagement for youth with SEBD. Intentional design of peer-inclusion models (segregated, inclusive, or reverse inclusive) to improve social support for PA among these youth can help not only improve PA engagement but several other long-term psychosocial outcomes (54).

## Discussion

The PAID Framework provides an approach to making meaningful and responsive PA program adaptations for youth with SEBD, taking into consideration their unique needs and family, social, and community contexts. Interventionists seeking to create programming for this population might make adaptations to the physical skills being learned and practiced, but without consideration of the youth’s prior experiences and potential negative perceptions, along with addressing the parental resources often required for successful engagement, efforts are likely to fail or not yield the desired outcomes.

As implied by the PAID Framework, it is critical that PA programs be culturally sensitive and appropriate. As discussed above, youth from minoritized populations experience difficulties accessing programming opportunities both as a result of their disability and their race, ethnicity, and/or other characteristics (63). Cultural adaptations address factors such as language, culture, and context in a manner that comports with program participants’ own cultural meanings and values (64). Several studies have documented that cultural adaptations promote the effectiveness of interventions through achieving a match between intervention components and participants’ cultural world views (65). Accordingly, programs must give consideration to participants’ cultural knowledge, values, norms, and traditions, and intervention staff must both understand and communicate such cultural information appropriately.

As suggested earlier, an inclusion team science approach, wherein experts in health-related programming work together with those with expertise in specific disabilities, helps ensure that programming meets the needs of the target population while maintaining evidence-based elements in the programming itself (in this case, PA) (25). A critical aspect of inclusion team science is the inclusion of individuals with lived experience in the development and pilot testing of program components. Including youth with SEBD to assess program feasibility, acceptability, and relevance is key to creating interventions that will have appeal, uptake, and ultimately efficacy and sustainability. Validated measures of acceptability and appropriateness completed by families such as the Acceptability of Intervention Measure (AIM) and Intervention Appropriateness Measure (IAM) can be used to assess goodness of fit and the need for additional modifications (66).

### Limitations

While we have sought to synthesize research in three broad areas to inform the PAID Framework, a systematic review of all the relevant literature was not possible given the breadth and interdisciplinary nature of the evidence base. Also, new health behavioral theories and implementation frameworks are continually being created and tested. For those reasons, the PAID Framework should be considered as an evolving framework into which additional theoretical or implementation constructs may be integrated over time. PAID should also change to reflect new knowledge about children and youth with SEBD and/or environmental exigencies. For example, the COVID-19 pandemic has highlighted the need for delivering interventions using remote means, but also highlighted how remote engagement may be inaccessible to low SES, at-risk families who are in greatest need of services. In many cases, remote participation in mental health services and health interventions has been viewed favorably and deemed preferable by youth and families, but disability accessibility is critical (67). Choosing in-person versus the use of remote or virtual technologies for program delivery platforms, including advantages and potential drawbacks, is another dimension that bears consideration as PA programming for youth are developed and implemented.

The PAID is a new PA engagement framework specifically for youth with SEBD; it can be used in part or in full both in the design of PA interventions *de novo* or as a guide for adapting existing PA programs for children and youth in the general population to meet the needs of youth with SEBD. The PAID Framework also provides elements that can be used to measure formative and summative work in the development and testing of PA interventions for children and youth with SEBD. Intervention designers and implementation scientists should consider using PAID as an integrated framework to increase PA programming engagement and decrease health disparities among youth with SEBD and their families, particularly among populations that have been historically missed in intervention reach.

## Author Contributions

AB and CC conceptualized, researched, and wrote the manuscript. JF, AS, and SB-F helped refine the framework, and substantially contributed to the development of the manuscript. All authors contributed to the article and approved the submitted version.

## Conflict of Interest

The authors declare that the research was conducted in the absence of any commercial or financial relationships that could be construed as a potential conflict of interest.

## Publisher’s Note

All claims expressed in this article are solely those of the authors and do not necessarily represent those of their affiliated organizations, or those of the publisher, the editors and the reviewers. Any product that may be evaluated in this article, or claim that may be made by its manufacturer, is not guaranteed or endorsed by the publisher.
